# Fighting Against Stroke in Latin America: A Joint Effort of Medical Professional Societies and Governments

**DOI:** 10.3389/fneur.2021.743732

**Published:** 2021-10-01

**Authors:** Sheila Cristina Ouriques Martins, Pablo Lavados, Thaís Leite Secchi, Michael Brainin, Sebastian Ameriso, Fernando Gongora-Rivera, Claudio Sacks, Carlos Cantú-Brito, Tony Fabian Alvarez Guzman, Germán Enrique Pérez-Romero, Mario Muñoz Collazos, Miguel A. Barboza, Antonio Arauz, Carlos Abanto Argomedo, Nelson Novarro-Escudero, Hector Ignacio Amorin Costabile, Roberto Crosa, Claudia Camejo, Ricardo Mernes, Nelson Maldonado, Daissy Liliana Mora Cuervo, Octávio Marques Pontes Neto, Gisele Sampaio Silva, Leonardo Augusto Carbonera, Ana Claudia de Souza, Eduardo David Gomes de Sousa, Alan Flores, Donoban Melgarejo, Irving R. Santos Carquin, Arnold Hoppe, João José Freitas de Carvalho, Francisco Mont'Alverne, Pablo Amaya, Hernan Bayona, Victor Hugo Navia González, Juan Carlos Duran, Victor C. Urrutia, Denizar Vianna Araujo, Valery L. Feigin, Raul G. Nogueira

**Affiliations:** ^1^Hospital Moinhos de Vento, Porto Alegre, Brazil; ^2^Hospital de Clínicas de Porto Alegre, Universidade Federal do Rio Grande do Sul, Porto Alegre, Brazil; ^3^Brazilian Stroke Network, Porto Alegre, Brazil; ^4^World Stroke Organization, Geneva, Switzerland; ^5^Clinica Alemana, Universidad del Desarollo, Santiago, Chile; ^6^Department of Clinical Neurosciences and Preventive Medicine, Danube University Krems, Krems an der Donau, Austria; ^7^Fundación para la Lucha contra las Enfermedades Neurológicas de la Infancia, Buenos Aires, Argentina; ^8^Servicio de Neurología - Unidad Neurovascular, Hospital Universitario José Eleuterio González, Universidad Autonoma de Nuevo Leon, Monterrey, Mexico; ^9^Instituto de Neurología y Neurocirugía, Centro Médico Zambrano Hellion, Tec Salud, San Pedro Garza García, Mexico; ^10^Centro de Investigación y Desarrollo en Ciencias de la Salud, Universidad Aiutónoma de Nuevo León, Monterrey, Mexico; ^11^Department of Neurology, Universidad del Valparaiso, Valparaiso, Chile; ^12^Department of Neurology, Instituto Nacional de Ciencias Médicas y Nutrición Salvador Zubiran, Mexico City, Mexico; ^13^Hospital Regional Manuela Beltran, Socorro, Colombia; ^14^Asociación Colombiana de Neurología, Bogotá, Colombia; ^15^Facultad de Medicina, Universidad Nacional de Colombia, Bogotá, Colombia; ^16^Fundación Hospital San Carlos, Bogotá, Colombia; ^17^Colombian Stroke Network, Bogota, Colombia; ^18^Hospital Dr. Rafael A. Calderon, Neuroscience Department, San José, Costa Rica; ^19^Instituto Nacional de Neurología y Neurocirugía Manuel Velasco Suárez, Clínica de Enfermedad Vascular Cerebral, Ciudad de México, Mexico; ^20^Departamento de Enfermedades Neurovasculares, Instituto Nacional de Ciencias Neurológicas, Lima, Peru; ^21^Pacífica Salud–Hospital Punta Pacífica, Panama City, Panama; ^22^Ministry of Health Uruguay, Montevideo, Uruguay; ^23^Médica Uruguaya, Montevidéo, Uruguay; ^24^Hospital das Clínicas, Montevideo, Uruguay; ^25^Hospital de Clinicas, Faculdad de Medicina, Universidad Nacional de Asuncion, San Lorenzo, Paraguay; ^26^Hospital Central del Instituto de Previsión Social, Asunción, Paraguay; ^27^Universidad San Francisco de Quito, Hospital de los Valles, Quito, Ecuador; ^28^Hospital das Clínicas da Faculdade de Medicina da Universidade de São Paulo, Ribeirão Preto, Brazil; ^29^Brazilian Stroke Society, São Paulo, Brazil; ^30^Department of Neurology and Neurosurgery, Universidade Federal de São Paulo, São Paulo, Brazil; ^31^Hospital Israelita Albert Einstein, São Paulo, Brazil; ^32^Ministry of Health, Brasília, Brazil; ^33^Emergency Hospital Public Assistance, Santiago, Chile; ^34^Faculty of Medicine, University of Chile, Santiago, Chile; ^35^Ministry of Health, Santiago, Chile; ^36^Facultad de Medicina, Universidad del Desarrollo, Santiago, Chile; ^37^Hospital Geral de Fortaleza, Fortaleza, Brazil; ^38^Sociedade Brazileira de Neurorradiologia Diagnóstica e Terapêutica, São Paulo, Brazil; ^39^Fundación Valle del Lili, Cali, Colombia; ^40^Fundación Santa Fé de Bogotá, Bogotá, Colombia; ^41^Sociedad Boliviana de Neurología, Santa Cruz, Bolívia; ^42^Department of Neurology, Johns Hopkins University School of Medicine, Baltimore, MD, United States; ^43^Faculdade de Ciências Médicas da Universidade do Estado do Rio de Janeiro, Rio de Janeiro, Brazil; ^44^National Institute for Stroke and Applied Neurosciences, School of Public Health and Psychosocial Studies, Faculty of Health and Environmental Sciences, Auckland University of Technology, Auckland, New Zealand; ^45^Marcus Stroke and Neuroscience Center, Grady Memorial Hospital, Emory University, Atlanta, GA, United States

**Keywords:** stroke, Latin America, stroke units, stroke centers, stroke system of care

## Abstract

**Introduction:** Stroke is one of the leading causes of death in Latin America, a region with countless gaps to be addressed to decrease its burden. In 2018, at the first Latin American Stroke Ministerial Meeting, stroke physician and healthcare manager representatives from 13 countries signed the Declaration of Gramado with the priorities to improve the region, with the commitment to implement all evidence-based strategies for stroke care. The second meeting in March 2020 reviewed the achievements in 2 years and discussed new objectives. This paper will review the 2-year advances and future plans of the Latin American alliance for stroke.

**Method:** In March 2020, a survey based on the Declaration of Gramado items was sent to the neurologists participants of the Stroke Ministerial Meetings. The results were confirmed with representatives of the Ministries of Health and leaders from the countries at the second Latin American Stroke Ministerial Meeting.

**Results:** In 2 years, public stroke awareness initiatives increased from 25 to 75% of countries. All countries have started programs to encourage physical activity, and there has been an increase in the number of countries that implement, at least partially, strategies to identify and treat hypertension, diabetes, and lifestyle risk factors. Programs to identify and treat dyslipidemia and atrial fibrillation still remained poor. The number of stroke centers increased from 322 to 448, all of them providing intravenous thrombolysis, with an increase in countries with stroke units. All countries have mechanical thrombectomy, but mostly restricted to a few private hospitals. Pre-hospital organization remains limited. The utilization of telemedicine has increased but is restricted to a few hospitals and is not widely available throughout the country. Patients have late, if any, access to rehabilitation after hospital discharge.

**Conclusion:** The initiative to collaborate, exchange experiences, and unite societies and governments to improve stroke care in Latin America has yielded good results. Important advances have been made in the region in terms of increasing the number of acute stroke care services, implementing reperfusion treatments and creating programs for the detection and treatment of risk factors. We hope that this approach can reduce inequalities in stroke care in Latin America and serves as a model for other under-resourced environments.

## Introduction

**Figure 1:**
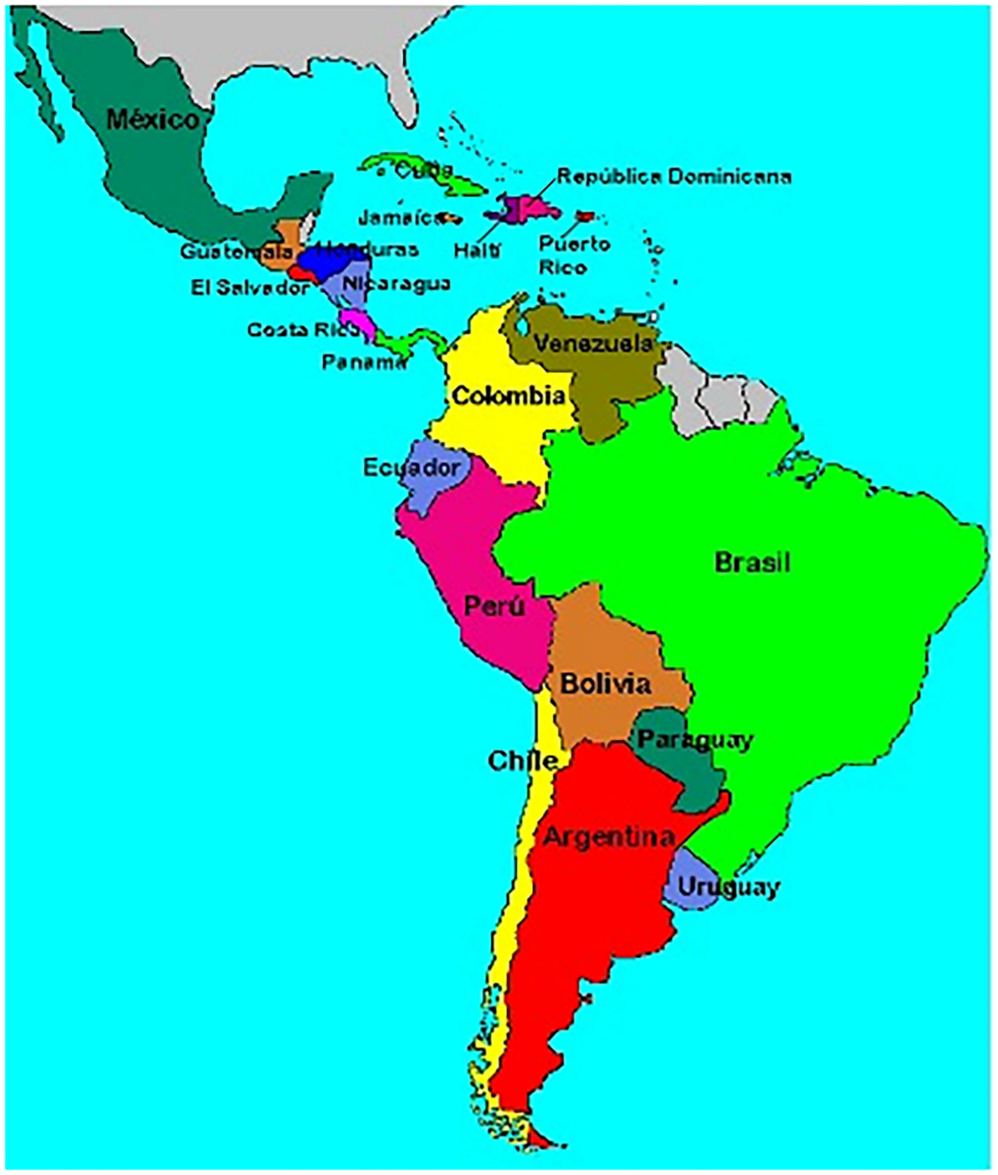
Map showing Latin American Countries.

Latin America consists of 20 countries on the American continent (Figure 1) which cover an area of 21 million km^2^, with an estimated population of 680 million inhabitants, with marked ethnic, cultural, and socioeconomic heterogeneity. A major development has occurred in the region in recent decades, with better control of communicable diseases. However, aging and population growth have led to an increase in the prevalence of cardiovascular risk factors, increasing mortality and disability due to stroke and cardiovascular diseases (1–4).

Stroke is the first or second leading cause of death in most countries in this region, with about 40% of these deaths occurring during a person's most productive years of their life (3, 4). Huge efforts have been made in recent years to improve stroke care in many Latin American countries led by stroke physicians. Nonetheless, there are still countless gaps to be addressed: access to stroke prevention and rehabilitation remains limited; most hospitals are not prepared for stroke care; few healthcare professionals are trained and available to assist acute stroke; there are few professional training and public awareness campaigns; funding for stroke programs, research, and education is scarce; and national stroke policies are established only in two countries (Brazil and Chile) (3).

In an initiative to join efforts to change this scenario, representatives of the Ibero-American Stroke Organization (SIECV), the World Stroke Organization and the American Heart Association/American Stroke Association organized a Latin American Stroke Ministerial Meeting in August 2018, during the pre-congress of the 21st Ibero-American Stroke Organization Congress in the city of Gramado, Brazil. The purpose of the Ministerial Meeting was to engage government leadership, healthcare policymakers, and civil society to explore and share promising efforts that were accelerating actions to address cerebrovascular diseases. It provided a forum to demonstrate how countries were meeting the Sustainable Development Goals (SDG) and continue to foster cooperation across the region to achieve the World Health Organization's goal of reducing pre-mature deaths from non-communicable diseases by one third by the year 2030 through prevention and treatment (SDG 3.4)^1^. The current status of the stroke burden in Argentina, Bolivia, Brazil, Chile, Colombia, Costa Rica, Ecuador, Guatemala, Mexico, Panama, Paraguay, Peru, and Uruguay was discussed, and the priorities to be addressed in this region of the world were defined with the signature of the Declaration of Gramado. The upshots of this meeting were published in Lancet Neurology (3).

In 2020, the Global Stroke Alliance^2^ was launched with the aim to stimulate a global alliance to improve stroke care around the world, with discussions on the best strategies to implement evidence-based interventions at all levels (prevention, treatment, and rehabilitation). At the first meeting in March 2020, in Rio de Janeiro, we gathered stroke leaders with large experience in the organization of stroke care systems, researchers, healthcare professionals, healthcare managers, and scientific societies representing 20 countries from all continents. An important part of the Global Stroke Alliance was the II Latin American Stroke Ministerial Meeting with the objectives of reviewing the achievements since the first meeting, making new commitments, and establishing deadlines for the implementation of stroke programs in the region. This article will review the 2-year advances and future plans of the Latin American alliance for stroke.

## Methods

In February 2020, we sent an electronic survey to the national leaders in stroke in Latin America to evaluate changes in the stroke care in their countries since the I Latin American Stroke Ministerial Meeting (August 2018). All of them participated in the previous meeting, and all were committed to improving the stroke program in their countries through evidence-based and cost-effective strategies.

We compared the 2020 survey results with the situation in 2018 published in Lancet Neurology as a result of the I Meeting (2). Data from 12 countries were available in 2020 (except Guatemala). The survey was based in the Declaration of Gramado items (Table 1) (3). Survey results were confirmed at a meeting in March 2020 with representatives of the Ministries of Health and country leaders of the 12 countries at the II Latin American Stroke Ministerial Meeting, in Rio de Janeiro. Also, the advances in stroke care over these 2 years in Latin American countries were evaluated at this meeting and will be reported in this paper.

**Table 1:** Declaration of Gramado.

**Declaration of Gramado** **Gramado, Brazil 2 August 2018**
**Commitments for Facing Stroke in Latin America countries:**
1. To provide education of the population on the stroke warning signs, treatment urgency, and control of risk factors;
2. To promote safe and healthy environments for the practice of physical activity;
3. To implement policies to control smoking, to stimulate healthy eating habits and physical activity, to reduce sodium intake and alcohol abuse and to control weight, aiming in reducing the incidence of cardiovascular and cerebrovascular diseases;
4. To set strategies for the detection of treatable risk factors, as hypertension, atrial fibrillation, diabetes, and hyperlipidemia;
5. To promote healthcare for the control of treatable risk factors;
6. To organize the prehospital care to prioritize the patient with stroke;
7. To prioritize the structuring of stroke centers:
• To organize stroke units with a defined physical space and a trained multidisciplinary team;
• To provide evidence-based acute treatments;
• To provide exams for minimal etiological workup;
• To promote the prescription of secondary prevention therapies in the hospital discharge;
• To encourage the use of telemedicine in hospitals without specialist 24 h a day, 7 days a week, to advise acute treatment.
8. To increase access to in-hospital and post-hospital rehabilitation;
9. To train all the professionals engaged in stroke care;
10. To monitor national prevalence of the main risk factors and quality indicators of stroke care nationwide;
11. To set national and regional evidence-based practice guidelines, with frequent updates, to standardize stroke care;
12. To prioritize the structuring of integrated networks for continuous care of patients with stroke or stroke risk factors, that encompass all levels of healthcare, creating a line of care;
13. To assign human and financial resources for the development of a stroke line of care;
14. To implement national stroke care policies;
15. To promote exchange of experiences among countries for the improvement of stroke care;
16. To implement research in stroke, based on the priorities and realities of each country.

To evaluate the number of stroke centers in the region, we considered a stroke center to be a hospital with a structure to assist stroke patients, including stroke team and reperfusion treatment (at least intravenous thrombolysis) available 24 h/day, 7 days/week (at least intravenous thrombolysis). Stroke unit was defined as a physical space within the stroke center solely devoted to assist stroke patients and staffed by a multidisciplinary team.

### Statistical Analysis

Frequencies were presented as proportions. Comparisons between categorical variables were made using χ^2^ or Fisher's exact-tests. A *p* of <0.05 was considered significant. Data were performed using SPSS software for Windows, version 20 (SPSS, Inc., Chicago, IL, USA).

## Results

The first actions to reduce the burden of stroke resulting from the I Latin American Stroke Ministerial Meeting in 2018 included: (I) publishing the results of the Ministerial Meeting with the Latin American countries priorities and commitments (3); (II) implementing a joint registry of quality indicators in stroke care; (III) structuring new stroke centers and encouraging the implementation of stroke units; and (IV) providing education to the population on stroke signs and participating in stroke campaigns of the World Stroke Organization. The publication of the Declaration of Gramado was an important landmark resulting from this event, in addition to the immediate adherence to the international quality indicators registries—Safe Implementation of Treatment in Stroke (SITS-QR)^3^ or Registry of Stroke Care Quality (RES-Q)^4^ —by the Latin American countries. In these 2 years, 190 hospitals in Latin America have participated in a joint registry of quality indicators, allowing for a continuous qualification of stroke centers. Cooperative work of hospitals, stroke neurologists, and governmental healthcare authorities increased during this period.

The 2020 survey was answered by 25 specialists from 12 countries. Answers from more than one specialist in each country were compared for consistency. In Table 2, we show the data from 2018 and 2020 on the implementation of National Stroke Policies, acute stroke care and rehabilitation from each country. National stroke policies were established in two countries in 2018 (Brazil and Chile) and are currently being implemented in Costa Rica. Uruguay, Paraguay, Colombia, and Mexico were working with their governments to establish a program for stroke.

**Table 2:** Stroke care in Latin American countries, 2018–2020.

**Country/income level/healthcare system**		**National plan for stroke**	**Acute stroke care**		**Rehabilitation**	
			Stroke centers^*^ and stroke units^**^	**Thrombectomy**	**In-hospital**	**After discharge**
**Argentina**/high income/100% MOH, 40% social security, 8% private	2018	No	1 public, 5 private stroke centers, no stroke units	Private	No data	No data
	2020	No	3 public−1 with stroke unit, 20 private stroke centers−5 with stroke units	5 private	Yes (private)	Yes
**Brazil/**upper middle/100% MOH/25% private	2018	Yes	156 stroke centers−50% private, 74 with stroke units	64 private, 2 public	Widely available in stroke units	Yes
	2020	Yes	182 stroke centers−50% private, 90 with stroke units	94 private, 4 public	Widely available in stroke units	Yes
**Bolivia/**lower middle/28% MOH/37% social security/10% private	2018	No	Few private stroke centers	Private	No data	No data
	2020	No	3 public stroke center, 4 private stroke centers	2 private	Yes (few public and private)	Yes
**Chile/**high/76% MOH/18% private	2018	Yes	20 public, 34 private stroke centers	6 public, 6 private	Widely available in stroke units	Yes
	2020	Yes	45 public, 34 private stroke centers 14 with stroke units	8 public, 12 private	Widely available in stroke units	Yes
**Colombia/**upper middle/48% public/45% private	2018	No	48 stroke centers, including private and public, no stroke units	Yes	Yes	No data
	2020	No	66 stroke centers, including 49 private and 17 public, 3 stroke units	3 public, 30 private	Yes (public and private)	Yes
**Costa Rica/**upper middle/90% public/10% private	2018	No	4 public, 1 private, all with stroke units	2 centers	Yes	Yes
	2020	Yes	4 public, 2 private, all with stroke units	4 centers	Yes (public and private)	Yes
**Ecuador/**upper middle/73% public, 27% private	2018	No	1 public center with stroke unit	No	Yes 1 hospital	No data
	2020	No	1 public, 1 private, both with stroke units	1 private	Yes (public and private)	Yes
**México/**upper middle/86% any coverage: 42% social security and 44% popular security (not cover stroke), 18% private	2018	No	2 public, 4 private stroke centers, 5 stroke units	Private	No data	No data
	2020	No	10 public, 30 private stroke centers, 8 stroke units	5 public, 10 private	Yes	Yes
**Panama/**high/90% (60–80% social security, 20–40% MOH), 5–10% private	2018	No	8 public, 5 private stroke centers	1 public, 4 private	Yes	Yes
	2020	No	8 public, 5 private stroke centers	1 public, 5 private	Yes (public and private)	Yes
**Paraguay/**upper middle/71% MOH, 21% social security, 7% private	2018	No	2 public stroke centers with stroke units, 6 other stroke centers	No	Yes	No data
	2020	No	2 public and 4 private, all with stroke units	2 public, 4 private	Yes	Yes
**Peru/**upper middle/51% MOH, 31% social security (EsSalud), 7% private	2018	No	4 public stroke centers−1 with stroke unit, 1 private stroke center	Yes (sporadic)	No data	No data
	2020	No	5 public stroke centers−4 with stroke units, 1 private stroke center	4 public, 1 private	Yes	Yes
**Uruguay/**high/37% public, 58% private	2018	No	18 centers, 1 public and 1 private with stroke units	1 private	No data	No data
	2020	No	18 centers, 1 public and 5 private with stroke units	1 public, 1 private	Yes	Yes

*MOH, Ministry of Health*.

* *A hospital with structure to assist stroke patients, including stroke team and reperfusion treatment available 24 h/day, 7 days/week (at least intravenous thrombolysis)*.

** *An exclusive geographic area inside the stroke center to assist stroke patients with a multidisciplinary team*.

The number of stroke centers increased from 322 to 448 during this period, in addition to a significant increase in stroke units. The highest increase in stroke centers was reported in Brazil, Chile, Colombia, and Mexico. Of the total, 44% of stroke centers were public. The highest increase in dedicated stroke units occurred in Brazil. Thrombectomy for patients with acute ischemic stroke was available in all countries in the 2020 survey, mainly in private hospitals. In-hospital rehabilitation services were improved in several hospitals, though access to post-discharge rehabilitation services remained limited.

In Table 3, we present the level of achievement of each country with the items of the Declaration of Gramado. Of the 12 countries, Chile and Brazil were the countries that most fulfilled the commitments made in 2018 in Gramado. Initiatives for public stroke awareness, including joining the World Stroke campaigns, increased in the period from 25% in 2018 to 75% in 2020 (*p* = 0.014). Despite this, only Chile, Costa Rica, and Peru have government programs for public awareness in stroke. Safe and healthy environments for the practice of physical activity have been implemented in all countries since 2018 (from 42 to 100%, *p* < 0.001) and policies to modify lifestyle risk factors that encourage healthy habits have been implemented in all countries, at least partially. Stroke Riskometer, an app for the population that evaluate their stroke risk at 5 and 10 years, identifies their own risk factors and teaches how to modify them, has been used by a small proportion of the population in 50% of the participating countries. The national program to detect and control vascular risk factors improved over the years in several countries (58% in 2018 to 92% in 2020, *p* = 0.06), mainly those focused on controlling hypertension and diabetes (75% of the countries), including free medication for the population. Smoking cessation and salt reduction programs have been implemented in 10 of the 12 countries. Healthcare polices for the diagnosis and treatment of risk factors have been created in several countries since 2018; however, there were still difficulties in the implementation of policies for detection and treatment of dyslipidemia (42% of the countries) or atrial fibrillation (all countries).

**Table 3:** Level of achievement of Latin American countries with the commitments of the Declaration of Gramado (2020).

**Commitments for Facing Stroke in Latin America countries: declaration of Gramado, Brazil, 2018**.	**Country**											
	**Argentina**	**Bolivia**	**Brazil**	**Chile**	**Colombia**	**Costa Rica**	**Ecuador**	**México**	**Panama**	**Paraguay**	**Peru**	**Uruguay**
Population education about stroke signs, treatment, and risk factor control	**∅**	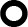	•	•	∅	•	◯	∅	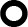	∅	•	∅
Safe and healthy environments for the practice of physical activity	•	•	•	•	•	•	•	•	•	•	•	•
Policies to control risk factors and to stimulate healthy habits	∅	∅	•	•	∅	∅	•	∅	∅	•	•	•
Strategies for the detection of treatable risk factors, as hypertension, atrial fibrillation, diabetes, and hyperlipidemia	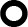	∅	∅	∅	∅	∅	∅	∅	∅	∅	∅	∅
Healthcare service for the control of modifiable risk factors	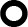	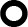	∅	•	∅	•	∅	∅	∅	∅	∅	•
Prehospital care to prioritize the patient with stroke	•	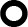	•	•	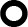	•	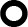	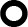	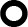	•	•	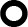
Stroke centers structure and stroke units	∅	∅	•	•	•	∅	∅	•	∅	∅	∅	∅
Access to in-hospital and post-hospital rehabilitation	∅	∅	∅	∅	∅	∅	∅	∅	∅	∅	∅	∅
Professionals trained in stroke care	∅	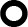	•	•	∅	•	∅	∅	∅	∅	∅	∅
National quality indicators of stroke care and control of risk factors	∅	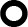	∅	•	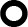	∅	∅	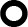	∅	∅	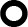	∅
National and regional evidence-based practice guidelines	•	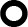	•	•	∅	•	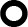	•	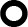	•	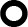	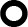
Integrated networks for continuous care of patients with stroke or stroke risk factors	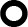	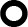	•	•	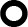	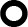	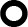	•	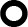	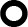	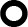	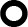
Human and financial resources for the development of a stroke line of care	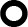	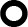	•	•	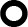	•	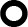	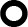	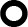	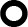	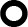	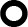
National stroke care policies	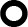	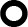	•	•	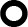	•	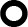	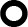	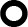	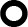	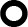	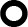
Exchange of experiences among countries for the improvement of stroke care	•	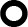	•	•	•	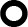	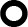	•	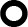	•	•	•
Research in stroke, based on the priorities and realities of each country	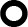	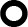	•	•	∅	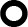	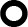	•	DK	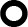	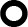	DK

*For indicators, the symbols represent the level of achievement: “•” fully achieved; “∅” partially achieved; “
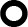
” not achieved; “DK” country responded “don't know”*.

**Figure 2:**
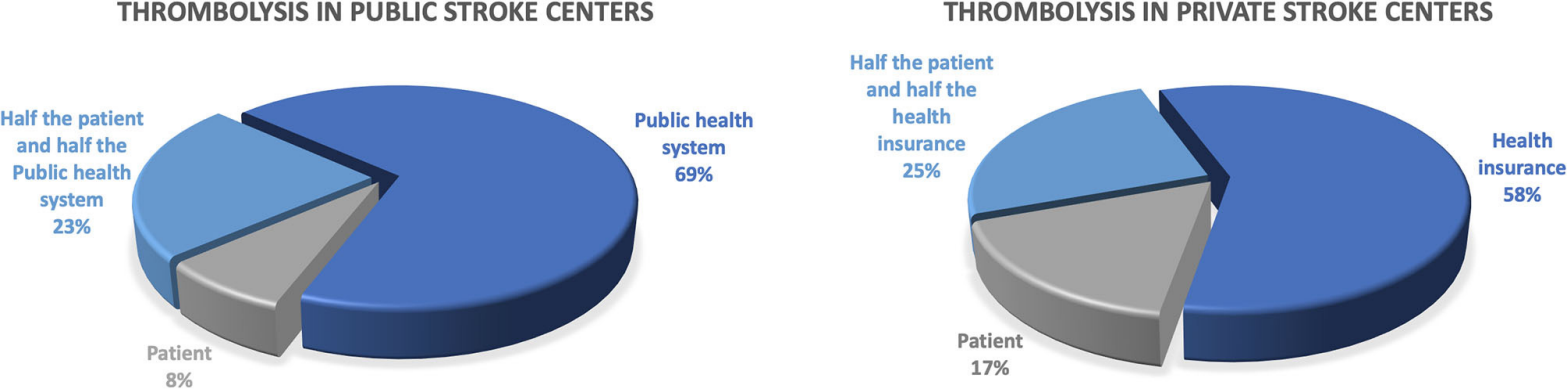
Who pays for intravenous thrombolysis?

**Figure 3:**
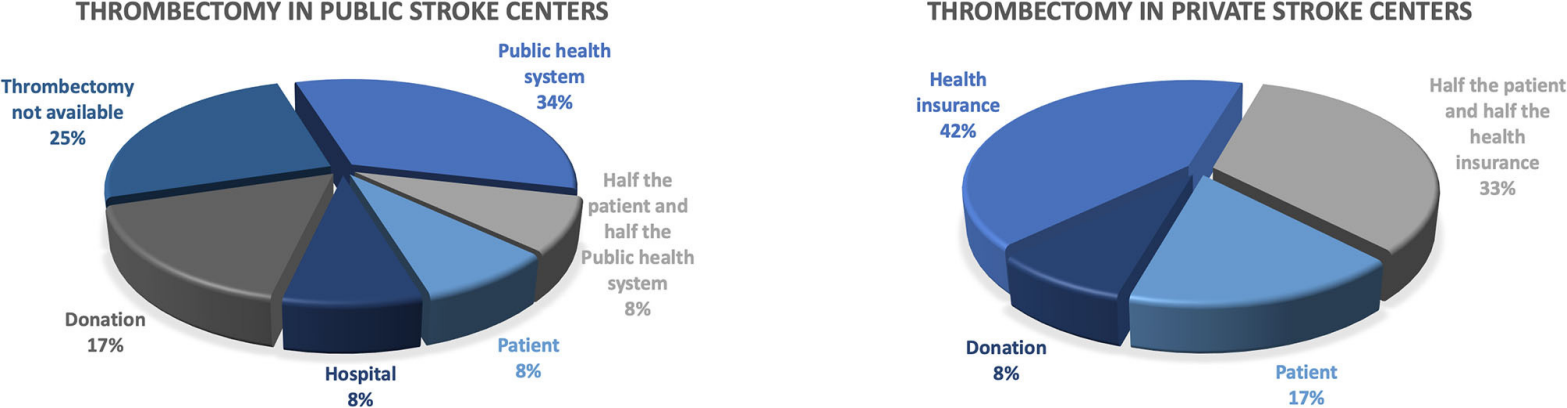
Who pays for mechanical thrombectomy?

Despite the improvement in the stroke centers structure, 58% of the countries reported that the pre-hospital care was not properly structured and the training of professionals for stroke care was limited, especially at the primary health care level. Five of the 12 countries reported having access to telemedicine in 2020 (42% compared with 17% in 2018, *p* = 0.43), but it remained restricted to a few hospitals and not widely available throughout the country. Figures 2, 3 describe the payer for intravenous thrombolysis and mechanical thrombectomy in public and private hospitals.

The research in stroke has increased in some countries. Brazil in recent years has had a major national advancement in clinical research, which has driven changes in public policies, such as the implementation of mechanical thrombectomy in the national public healthcare system supported by the result of RESILIENT trial. Chile, México, and Colombia also reported an increase in the national incentive for stroke research since 2018 but with still limited resources.

Importantly, most countries (92%) reported improvement in stroke care in this period between 2018 and 2020. The implementation of policies to control risk factors and encourage healthy habits, the structuring of stroke centers and the increase availability of intravenous thrombolysis and thrombectomy in acute stroke were the most cited.

## Discussion

Despite the great impact of stroke in Latin American countries, the initiative to collaborate, exchange experiences, and unite all the stakeholders including the governments has yielded good results. Important advances have been made in the region in terms of increasing the number of services for acute stroke care, implementing acute phase treatments (thrombolysis and thrombectomy) and creating programs for the detection and treatment of hypertension, diabetes, and lifestyle modification, essential strategies recommended by the World Health Organization^1^ and World Stroke Organization (5–7).

Since 2018, the Declaration of Gramado has been a guide to be followed by the Latin American countries for all evidence-based strategies to improve stroke care, and the implementation of recommended strategies has shown a significant improvement in the region in this short period of time. Chile and Brazil, both with National Policies since 2007 and 2012, respectively^5^^,^^6^^,^^7^, had the highest number of recommendations implemented. The National Stroke Policy being implemented in Costa Rica was another important achievement in this period.

The awareness campaigns launched by the World Stroke Organization^8^ in 2006 have being increasingly used every year. In Latin American countries, the improvement was significant in the number of countries with national campaigns (from 25 to 75%) as well as in the number of governmental and non-governmental initiatives. It is necessary to measure the impact of these campaigns on the population's knowledge about stroke. Although a study has shown better recognition of risk factors in recent years in Argentina (8, 9), recognition of warning signs remains very low and actions that could save lives and reduce the inability to call an ambulance and go to a stroke center are still very scarce (8, 10, 11).

Despite the great international appeal for the implementation of effective and low-cost strategies in primary prevention (12–16), such as reducing salt intake, encouraging physical exercise and weight reduction, discontinuing alcohol and unhealthy foods intake, in our study, as in many countries across the globe (17–19), there has been only a partial implementation of these strategies that could have a huge impact in decreasing the global burden of cardiovascular diseases (CVD). An important aspect is that 100% of Latin American countries in our study encouraged the physical activity, which could reduce the risk of CVD and help to control obesity, reducing blood pressure and cholesterol. In addition, 100% of countries had adhered to some smoking cessation strategy, including countries that are models for this proposal, such as Brazil and Panama (3). Knowledge about stroke prevention has advanced a lot in recent years, but the strategies for implementation have failed to reduce the burden (6, 17). Free motivational tools as Stroke Riskometer have shown the potential to help in lifestyle modification and could be an opportunity to increase education about risk factors and how to control them (5, 20–23). The Stroke Riskometer has been used by part of the population in 50% of the Latin American countries and increasing its use could help to control risk factors.

Detection and control of the cardiovascular risk factors, such as hypertension and diabetes, has greatly improved over these 2 years, including the availability of free drugs in 75% of the evaluated countries. Dyslipidemia was not adequately monitored and treated. Atrial fibrillation has not been screened in many countries, probably based on the fact that the benefit of screening in primary prevention has not been demonstrated. Furthermore, there have been no treatment policies in the region despite the fact that detection of atrial fibrillation and appropriate treatment could reduce the risk of stroke by 64% in patients with this neglected risk factor, especially in low- and middle-income countries (LMIC) (24, 25).

The huge adherence to the international quality indicator registries (RES-Q and SITS-QR) from zero in 2018 to 190 hospitals in 2 years is a good example of how this type of collaboration can lead to greater motivation to implement new changes. The World Stroke Organization/Ibero-American Stroke Organization Angels Award, implemented in August 2018 to recognize and honor teams and individuals committed to improving the quality of stroke practice, was an important way to establish a culture of continuous monitoring, acknowledging those who achieve the benchmarks in the quality indicators for stroke care. The Angels Initiative^9^, with a team of consultants, was also a very important tool to identify capable hospitals and help them in the first steps toward the implementation of stroke centers. They were also important in the qualification of stroke centers that were already established, identifying the gaps to the reperfusion treatment delay and providing guidance to reduce these gaps.

There was a 35% increase in the number of stroke centers in the region in 2 years and an increase in stroke units (67% of countries in 2018 to 83% of countries in 2020). The greater increase occurred in Brazil because of the National Stroke Program with better reimbursement for hospitals with stroke units^3^^,^^4^. Stroke units are a highly effective strategy in acute care and early rehabilitation to reduce stroke morbidity and mortality (26) and their implementation should be a priority in Latin America. Unfortunately, they are underutilized or unused, as shown in a recent paper that evaluate stroke services across the world (27), with the worst situation in LMIC.

Despite the increase in stroke centers in the period, pre-hospital stroke care remains limited, as 58% of the countries did not have an organized system. Pre-hospital care is a fundamental part of the organization of stroke systems of care to ensure early arrival at the prepared hospital and the better outcomes (28).

The implementation of mechanical thrombectomy occurred in a small number of hospitals in all countries and remains mostly restricted to private hospitals. Chile has had thrombectomy as a government policy with official reimbursement to public hospitals since January 2020^2^ (29). The RESILIENT trial (30), a randomized thrombectomy trial across stroke centers in the Brazilian Public Healthcare System, demonstrated the safety, efficacy, and cost-effectiveness of this treatment in under-resourced settings. Since the study results in 2019, the number of public hospitals adopting thrombectomy has been continuously increasing in Brazil. We expect an even higher impact in the current year with the recent incorporation of thrombectomy as a reimbursed treatment by the Brazilian Ministry of Health. This study is a good example of how government-funded research can lead to significant changes in public healthcare policy. Additional collaborative research efforts are in place, generating a good source of information about stroke in the region with potential global implications.

Regardless of the increasing number of stroke centers in the region, in some countries the patient must pay for reperfusion therapies, at least in part. In public hospitals, intravenous thrombolysis needs full payment by the patient in 8% of countries whereas in 23% of the countries half of the costs are paid by the patient and the other half by the government. In private hospitals, intravenous thrombolysis in 42% of the countries are not fully covered by private insurance and the patient pays for treatment in 17% of countries and half the cost in 25% of countries. Non-reimbursement of thrombolytic medication certainly limits access for several patients. Discussions with healthcare managers to reinforce that the treatment has been level 1A of evidence since 1995 (31), is effective and cost-saving (32) and that implementation has a high impact on patient outcomes (33), decreasing disability and costs, could help to change this scenario. For mechanical thrombectomy (MT), the situation is even worse. In private hospitals, MT is fully paid by private insurance in only 42% of countries. In public hospitals, the government pays for MT in only 35% of countries.

Telemedicine is a low-cost tool used to increase access to stroke centers and stroke experts, including smartphone mobile apps validated in the region (34). Unfortunately, despite of the increment from 17% in 2018 to 42% in 2020, it was restricted to a few hospitals and poorly distributed in several countries. A similar situation occurs around the world with higher availability of telemedicine in high-income countries, where the population already has access to better organized stroke care and specialists (27).

Adequate and accessible rehabilitation is still a problem in most countries, with 92% of them indicating difficulty of accessibility of community rehabilitation after hospital discharge. The service might even be available, but the patients only get access months after stroke onset, missing out the most important period for stroke recovery. The situation is similar in other LMIC countries (27). The training of healthcare professionals remains limited, especially in primary care, which is the critical step in the detection and monitoring of cardiovascular risk factors.

Our study has limitations. It is based on a survey of expert opinion on stroke, but it is from country leaders with large experience in the stroke field, working to improve stroke care, several of them in close contact with the healthcare managers and governments. Furthermore, the results were discussed at the Ministerial Meeting together with the Ministry of Health representatives to confirm the findings. In addition, this study is important because it assesses not only the aspects that have improved but also the greatest difficulties in the region that need to be address immediately.

### Next Steps

The II Ministerial Meeting took place in March 11th, 2020, at the global inception of the coronavirus pandemic. Despite the hectic global situation and the constraints on controlling the pandemic, Latin American leaders continued their efforts to structure the centers and promote education on stroke, through initiatives such as the Global Stroke Alliance—the fight must go on, a free virtual platform that integrated the professionals with a high-quality program. Weekly virtual meetings included the planning and implementation of the WSO/SIECV Certification of Stroke Centers in Latin America, launched in March 2021. The Certification goal is to ensure the implementation of all evidence-based strategies in stroke care with a strong quality program, with online application and pre-certification, followed by an onsite visit and a final international board review to complete the process. There are currently have 24 hospitals in the process of certification, with the first on-site occuring in July 2021. It is a great opportunity for continuous improvement of services and qualification of stroke care in the region and a priority step to guide the national stroke care along evidence-based pathways.

Also, an important step is the implementation in several Latin American countries of the HEARTS Initiative, a WHO program to decrease cardiovascular events through the control of hypertension, diabetes, and lifestyle modifications. The program is based on a gradual, slow, and monitored change in the assistance in primary care, focusing on flowcharts and simplification of processes through protocols. The program is being implemented in Argentina, Bolivia, Brazil, Chile, Colombia, Dominican Republic, Ecuador, Mexico, Panama, and Peru and has been widely discussed at the Global Stroke Alliance platform with support of the Pan-American Health Organization (PAHO, which represents the WHO in the Americas)^10^, the Ministry of Health of Brazil, and the World Stroke Organization to implement the HEARTS program in Brazil, to treat patients with hypertension and diabetes, and to implement a clinical trial to assess the concept of treating patients at middle risk of stroke (systolic blood pressure 121–139 mmHg plus one lifestyle risk factor) in primary care using an antihypertensive plus statin polypill, Stroke Riskometer and working with community health workers. Our goal is to demonstrated a 50% reduction in stroke incidence with this program (the WSO “Cut stroke in half” Initiative) (35).

## Conclusions

The implementation of evidence-based public policies is essential for the prevention and treatment of stroke and its risk factors. The collective work of governments and professional medical societies is necessary to address the catastrophic effects of stroke. In Latin America, the strength of our regional stroke network allowed us the opportunity to learn from each other and improve our prevention strategies, our data quality, and the development of our stroke centers. We hope that this approach can reduce inequalities in stroke care in the region, decreasing the gaps between public and private systems, rich and poor people, and among low-, middle-, and high-income countries.

## Data Availability Statement

The raw data supporting the conclusions of this article were made available by the authors, without undue reservation.

## Author Contributions

SM analyzed the data, prepared the first draft of the manuscript, and reviewed all drafts. TS analyzed data reviewed and edited the first draft and final versions of the manuscript. PL, MB, SA, FG-R, CS, CC-B, TA, GP-R, MM, MAB, AA, CA, NN-E, HA, RC, CC, RM, NM, DLM, OP, GS, LC, AS, ES, AF, DM, IS, AH, JC, FM'A, PA, HB, VN, JD, VU, DA, VF, and RN provided data, reviewed the results, and approved the final version of the manuscript. All authors contributed to the article and approved the submitted version.

## Conflict of Interest

The authors declare that the research was conducted in the absence of any commercial or financial relationships that could be construed as a potential conflict of interest.

## Publisher's Note

All claims expressed in this article are solely those of the authors and do not necessarily represent those of their affiliated organizations, or those of the publisher, the editors and the reviewers. Any product that may be evaluated in this article, or claim that may be made by its manufacturer, is not guaranteed or endorsed by the publisher.

## Footnotes

^1^ https://www.who.int/health-topics/sustainable-development-goals#tab=tab_2

^2^ http://www.globalstrokealliance.com/en/home-2/

^3^ https://sitsinternational.org/

^4^ https://qualityregistry.eu

^5^ https://redcronicas.minsal.cl/ataque-cerebrovascular/

^6^ http://189.28.128.100/dab/docs/portaldab/publicacoes/LC_AVC_no_adulto.pdf

^7^ https://bvsms.saude.gov.br/bvs/saudelegis/gm/2012/PRT0665_12_04_2012.html

^8^ https://www.world-stroke.org/world-stroke-day-campaign/world-stroke-campaign

^9^ https://www.angels-initiative.com

^10^ https://www.paho.org/en/hearts-americas
