# Supplementary material for: Fighting Against Stroke in Latin America: A Joint Effort of Medical Professional Societies and Governments
Source: Front Neurol. 2021 Oct 1;12:743732. doi: 10.3389/fneur.2021.743732 (PMC8517273; doi:10.3389/fneur.2021.743732)
Supplement: Supplementary file 1 [file Data_Sheet_1.docx]

| Stroke Care in Latin America – Survey 2020 |
| --- |
| 1. Does your country have a national stroke program? |
| [Yes] [No] [Do not know] |
|  |
| 1. Does your country have government policies to: |
|  |
| 1. educate the population about stroke? [Yes] [No] [Do not know] |
| 1. stimulate physical activity? [Yes] [No] [Do not know] |
| 1. control smoking? [Yes] [No] [Do not know] |
| 1. encourage healthy eating? [Yes] [No] [Do not know] |
| 1. reduce salt intake? [Yes] [No] [Do not know] |
| 1. reduce harmful alcohol consumption? [Yes] [No] [Do not know] |
| 1. control the weight? [Yes] [No] [Do not know] |
|  |
| 1. Does your country have government policies for the DETECTION of risk factors? |
|  |
| 1. Hypertension [Yes] [No] [Do not know] |
| 1. Diabetes [Yes] [No] [Do not know] |
| 1. Dyslipidemia [Yes] [No] [Do not know] |
| 1. Atrial fibrillation [Yes] [No] [Do not know] |
|  |
| 1. Does your country have government policies for the TREATMENT of risk factors? |
|  |
| 1. Hypertension [Yes] [No] [Do not know] |
| 1. Diabetes [Yes] [No] [Do not know] |
| 1. Dyslipidemia [Yes] [No] [Do not know] |
| 1. Atrial fibrillation [Yes] [No] [Do not know] |
| 1. Smoke [Yes] [No] [Do not know] |
|  |
| 1. Is there FREE access to preventive medications for the following illnesses? |
|  |
| 1. Hypertension [Yes] [No] [Do not know] |
| 1. Diabetes [Yes] [No] [Do not know] |
| 1. Dyslipidemia [Yes] [No] [Do not know] |
| 1. Atrial fibrillation [Yes] [No] [Do not know] |
| 1. Smoke [Yes] [No] [Do not know] |
|  |
| 1. Is the Stroke Riskometer used in your country? |
|  |
| [Yes, most of the population] |
| [Yes, a moderate part of the population] |
| [Yes, a small part of the population] |
| [No] |
| [Do not know] |
|  |
| 1. Are there other non-governmental educational initiatives for the population? |
|  |
| [Yes, nationwide] |
| [Yes, local] |
| [No] |
| [Do not know] |
|  |
| 1. Is the pre-hospital care organized, giving priority to stroke patients? |
| [Yes] [No] [Do not know] |
|  |
| 1. Is the pre-hospital care organized for stroke public or private? |
| [Public] [Private] [Public and private] [Do not know] |
|  |
| 1. Does your country have government policies for the IMPLEMENTATION of stroke centers? |
| [Yes] [No] [Do not know] |
|  |
| 1. How many public stroke centers are there in your country? |
|  |
| 1. Do public stroke centers have: |
|  |
| 1. stroke units with a defined physical area and a trained multidisciplinary team? |
| [Yes, always] [Yes, sometimes] [No] [Do not know] |
|  |
| 1. reperfusion treatment with intravenous thrombolysis? |
| [Yes, always] [Yes, sometimes] [No] [Do not know] |
|  |
| 1. reperfusion treatment with mechanical thrombectomy? |
| [Yes, always] [Yes, sometimes] [No] [Do not know] |
|  |
| 1. tests for basic investigation of stroke etiology? |
| [Yes, always] [Yes, sometimes] [No] [Do not know] |
|  |
| 1. hospital discharge with prescription of stroke secondary prevention? |
| [Yes, always] [Yes, sometimes] [No] [Do not know] |
|  |
| 1. telemedicine in hospitals without access to a full-time specialist to guide acute stroke care? |
| [Yes, always] [Yes, sometimes] [No] [Do not know] |
|  |
| 1. How many public centers have stroke units? |
|  |
| 1. If public centers have thrombolysis, it is paid by: |
|  |
| [Public system] |
| [Patient] |
| [Half the patient and half the public system] |
| [Other] |
| [Do not know] |
|  |
| 1. How many public hospitals have thrombectomy in your country? |
|  |
| 1. If public centers have thrombectomy, it is paid by: |
|  |
| [Public system] |
| [Local public health authorities] |
| [Hospital] |
| [Patient] |
| [Half the patient and half the public system] |
| [Other] |
| [Do not know] |
|  |
| 1. Does your country have private stroke centers? |
|  |
| 1. How many private stroke care centers are there in your country? |
|  |
| 1. Do private stroke centers have: |
|  |
| a) stroke units with a defined physical area and a trained multidisciplinary team? |
| [Yes, always] [Yes, sometimes] [No] [Do not know] |
|  |
| 1. reperfusion treatment with intravenous thrombolysis? |
| [Yes, always] [Yes, sometimes] [No] [Do not know] |
|  |
| 1. reperfusion treatment with mechanical thrombectomy? |
| [Yes, always] [Yes, sometimes] [No] [Do not know] |
|  |
| 1. tests for basic investigation of stroke etiology? |
| [Yes, always] [Yes, sometimes] [No] [Do not know] |
|  |
| 1. hospital discharge with prescription of stroke secondary prevention? |
| [Yes, always] [Yes, sometimes] [No] [Do not know] |
|  |
| 1. telemedicine in hospitals without access to a full-time specialist to guide acute stroke care? |
| [Yes, always] [Yes, sometimes] [No] [Do not know] |
|  |
| 1. How many private centers have stroke units? |
|  |
| 1. If private centers have thrombolysis, it is paid by: |
|  |
| [Private health insurance] |
| [Social insurance |
| [Patient] |
| [Half the patient and half the private health insurance] |
| [Other] |
| [Do not know] |
|  |
| 1. How many private hospitals have thrombectomy in your country? |
|  |
| 1. If private centers have thrombectomy, it is paid by: |
|  |
| [Private health insurance] |
| [Social insurance |
| [Patient] |
| [Half the patient and half the private health insurance] |
| [Other] |
| [Do not know] |
|  |
| 1. How many hospitals use telemedicine in stroke care? |
|  |
| 1. If your country uses telemedicine, it is used to: |
|  |
| [Guide thrombolysis] |
| [Evaluate the case and guide the transfer to a stroke center for thrombolysis] |
| [Guide the transfer to thrombectomy] |
| [Other] |
|  |
| 1. Do stroke centers have hospital rehabilitation? |
|  |
| 1. Public [Yes, always] [Yes, sometimes] [No] [Do not know] |
| 1. Private [Yes, always] [Yes, sometimes] [No] [Do not know] |
| 1. Other [Yes, always] [Yes, sometimes] [No] [Do not know] |
|  |
| 1. Does your country have rehabilitation after hospital discharge? |
|  |
| [Yes, with fast access after hospital discharge] |
| [Yes, with difficult access after hospital discharge] |
| [No] |
| [Do not know] |
|  |
| 1. In your country, the training of health professionals to care for stroke patients, in general: |
|  |
| 1. In the pre-hospital [Very good] [Good**]**  [Reasonable] [Bad] [Very bad] |
| 1. In public hospitals [Very good] [Good**]** [Reasonable] [Bad] [Very bad] |
| 1. In private hospitals [Very good] [Good**]** [Reasonable] [Bad] [Very bad] |
| 1. In primary care [Very good] [Good**]** [Reasonable] [Bad] [Very bad] |
|  |
| 1. In your country, is there national monitoring of the prevalence of the main risk factors? |
|  |
| 1. In your country, is there a National monitoring of hospital care indicators for stroke care? |
|  |
| 1. Are there national and regional guidelines based on scientific evidence for standardized stroke care with periodic updates? |
|  |
| 1. Does your country prioritize the structuring of integrated care networks for the continuity of care for patients with stroke that encompass all levels of care, establishing a Line of Care? |
|  |
| 1. Does your country allocate human and financial resources for structuring the Stroke Care Line? |
|  |
| 1. Does your country allocate resources for research into stroke based on the priorities and realities of the country? |
|  |
| 1. Are there exchanges of experiences between countries to improve stroke assistance? |
|  |
| 1. Do you think stroke care has improved in your country in the last 2 years? |
|  |
| 1. Is there any new initiative in the country in relation to stroke, not mentioned in this survey? Please describe. |
|  |
